# Influenza H7N9 virus disrupts the monolayer human brain microvascular endothelial cells barrier in vitro

**DOI:** 10.1186/s12985-023-02163-3

**Published:** 2023-09-29

**Authors:** Yuxuan Lei, Ying Sun, Weihua Wu, Hui Liu, Xin Wang, Yuelong Shu, Shisong Fang

**Affiliations:** 1 School of Public Health (Shenzhen), Sun Yat-sen University, Shenzhen, 518107 China; 2 Shenzhen Center for Disease Control and Prevention, Shenzhen, 518055 China; 3 Institute of Pathogen Biology, Chinese Academy of Medical Sciences and Peking Union Medical College, Beijing, 100730 China

**Keywords:** Influenza, Influenza H7N9 virus, hCMEC/D3 cells, The blood-brain barrier

## Abstract

Influenza H7N9 virus causes human infections with about 40% case fatality rate. The severe cases usually present with pneumonia; however, some present with central nervous system complications. Pneumonia syndrome is attributed to the cytokine storm after infection with H7N9, but the pathogenic mechanism of central nervous system complications has not been clarified. This study used immortalized human brain microvascular endothelial cells hCMEC/D3 to simulate the blood-brain barrier. It demonstrated that H7N9 virus could infect brain microvascular endothelial cells and compromise the blood-brain barrier integrity and permeability by down-regulating the expression of cell junction-related proteins, including claudin-5, occludin, and vascular endothelial (VE)-cadherin. These results suggested that H7N9 could infect the blood-brain barrier in vitro and affect its functions, which could be a potential mechanism for the pathogenesis of H7N9 viral encephalopathy.

**Supplementary Information:**

The online version contains supplementary material available at 10.1186/s12985-023-02163-3.

## Background

Influenza belongs to orthomyxoviridae, an enveloped segmental negative-strand RNA virus [1]. According to the antigenicity difference of nuclear protein (NP) and matrix protein (M), influenza viruses can be divided into A, B, C and newly discovered type D influenza virus [2]. Among them, influenza A virus (IAV) infects a wide range of hosts, which can not only infect a variety of mammals and birds but also infect humans across interspecies barriers [3]. Due to the lack of proofreading activity of polymerase and segmented genome, the antigenic drift and shift cause the virus diversity and the emergence of novel IAV [4]. The H7N9 influenza A virus was first isolated in 2013, composed of early H7N9, H7N3 and H9N2 influenza virus gene segments and continued to cause human infections [5, 6]. The emergence of the highly pathogenic H7N9 influenza virus has seriously threatened poultry production and human health [7]. In particular, the emergence of highly pathogenic H7N9 virus variants and the ability of limited human-to-human transmission need more attention.

The typical clinical symptoms of influenza are cough, high fever, muscle pain, and general discomfort, but some influenza patients have nervous system symptoms such as febrile seizures, encephalitis/encephalopathy, and myelitis [8, 9]. Central nervous system complications caused by influenza virus infection have been reported frequently[8, 10–13]. According to previous case reports and studies, Influenza-associated neurological complications often occur in children, and most cases are caused by influenza A and B viruses [11, 14, 15]. Neurological complications caused by influenza virus infection have gradually attracted more attention, but its pathogenic mechanism remains unclear [11–13, 15, 16]. Whether the influenza virus can invade the central nervous system (CNS) is controversial. However, some laboratory evidence suggested that IVA could infect the CNS. In both ferret and mouse models, H5N1 could reach the CNS through multiple pathways, such as the olfactory, vagus, and vestibulocochlear nerves [17, 18]. HPAI virus H7N1 could destroy the blood-brain barrier and lead to viremia and pathological changes in the central nervous system of chickens [19]. Besides, In vivo studies have shown that H7N9 virus RNA was detected in the brains of experimentally infected ferrets and mice [20, 21], suggesting that avian influenza H7N9 virus might spread to the brains of mammals.

It has been reported that H7N9 virus could cause neurological manifestations in patients [22, 23]. Besides, human astrocytic and neuronal cells could be infected by H7N9 virus, and viral infection triggered high expression of pro-inflammatory cytokines [24]. However, there is no enough experimental data to indicate how H7N9 virus enters the CNS. Considering previous studies and viremia in avian influenza virus infection [25], the blood pathway could be the potential route of the H7N9 avian influenza virus entering the CNS. H7N9 virus needs to pass the blood-brain barrier to reach the CNS. The blood-brain barrier (BBB) is a dynamic regulator of ion balance, a facilitator of nutrient transport and a barrier to potentially harmful molecules that acts as an interface between the central nervous and peripheral circulatory systems [26, 27]. BBB mainly comprises microvascular endothelial cells, pericytes, astrocytes and basement membrane [28]. The core element of the BBB is the cerebral vasculature formed by endothelial cells, which build a physical barrier between the blood and the brain. Increased endothelial cell permeability leads to BBB disruption and is a hallmark of CNS infection [29]. In this study, immortalized human brain microvascular endothelial cells hCMEC/D3 were used to construct an in vitro BBB model to explore whether H7N9 virus could destroy the BBB for the pathogenesis of influenza viral encephalopathy.

## Methods

### Cell culture

The immortalized human brain capillary endothelial cell line hCMEC/D3 purchased from BeNa Culture Collection was cultured in endothelial cell medium (ECM) supplemented with 5% fetal bovine serum (FBS), 1% endothelial cell growth supplement and 1% penicillin/streptomycin (P/S). The Madin-Darby canine kidney cell line MDCK was cultured in a Dulbecco’s modified eagle medium (DMEM) supplemented with 10% FBS and 1% P/S. Both cells were incubated at 37℃ with 5% CO2 and used from passage 8–20.

### H7N9 virus infection

The virus A/Shenzhen/13/2013 (H7N9), kindly provided by the Shenzhen Center for Disease Control and Prevention, was propagated in SPF eggs. For virus infection assays, hCMEC/D3 cells were seeded on 6-well plates (5 × 10^5^ cells/well) and incubated until cells were grown to 80-90% confluent monolayer. Cells were then infected with H7N9 virus at different multiplicities of infection (MOI). For the mock group, egg allantoic fluid without virus was added into the medium after cells were grown to 80-90% confluent monolayer. During infection, cell morphology was photographed every 24 h until 48 h after infection and supernatant was collected simultaneously to detect the progeny virus titer at each time point. All infection experiments were carried out in the class III bio-safety lab (BSL-3) at Shenzhen Center for Disease Control and Prevention.

### Virus titer assay

The 50% cell culture infectious dose (TCID50) endpoint dilution assay was performed for the virus titer. MDCK cells were inoculated into a 96-well cell plate (2 × 10^4^ cells/well) and incubated at 37℃ with 5% CO2 for 24 h, and the virus was diluted in a 10-fold gradient with medium containing 2 µg/mL TPCK-trypsin. When the cells reached 90% density, PBS washed the cells twice. The virus diluted as described above was inoculated on the 96-well plate containing MDCK cells, four wells per dilution, incubated at 37℃ for 1 h. After incubation, the medium containing the virus was discarded, and the cells were washed with PBS once. The culture medium containing 2 µg/mL TPCK-trypsin was added to each well and incubated at 37℃ for 72 h to detect the virus’s erythrocyte agglutination (HA) titer. The TCID50/100µL of the virus was calculated according to Reed-Muench methods (Table 1).

### Quantitative real-time PCR

Quantitative real-time PCR (qPCR) was performed to detect the presence of the viral genome in hCMEC/D3 cells and the transcript level of tight junctional complexes. The infected cells were collected at 2 h, 24 h, 48 h, and 72 h after infection, and the total RNA was extracted using the AxyPrep Multisource Total RNA Miniprep Kit (Axygen). PCR amplification was performed using the One Step TB Green PrimeScript RT-PCR Kit II (Takara).

**Table 1:** The primers used in qPCR

Genes	Forword primers	Reverse primers
Flu A	GACCAATCCTGTCACCTCTGAC	AGCTGAGTGCGACCTCCTTAG
Claudin 5	CTCTGCTGGTTCGCCAACAT	CAGCTCGTACTTCTGCGACA
Occludin	ACAAGCGGTTTTATCCAGAGTC	GTCATCCACAGGCGAAGTTAAT
VE-cadherin	TTGGAACCAGATGCACATTGAT	TCTTGCGACTCACGCTTGAC
*β*-actin	CTCCATCCTGGCCTCGCTGT	GCTGTCACCTTCACCGTTCC

The table shows all the primers used for qPCR, and the primers are written in the order from 5′ to 3′

### Cell viability assay

In order to detect the cell viability of hCMEC/D3 cells after infection, a CCK-8 test was carried out. HCMEC/D3 cells were seeded on a 96-well plate (1.5 × 10^4^/well) and incubated until cells were grown to 80% confluent monolayer. PBS washed cells once, and 200µL medium containing 2% FBS was added to each well. Then cells were infected with H7N9 influenza virus at different MOIs. 24 h, 48 and 72 h after infection, the number of living cells was detected by CCK-8 kit (Beyotime). The following formula calculated the cell viability:

$${Cell} \; {viability} = \frac{{OD450}_{H7N9}}{{OD450}_{Mock}}$$

### Western blot analysis

To detect the effect of virus infection on tight junction proteins in endothelial cells, cells were lysed in RIPA buffer (Beyotime). Proteins were separated by 10% SDS-PAGE and transferred to polyvinylidene fluoride membranes which were blocked with a blocking buffer (3% bovine serum albumin (BSA) in TBS with 0.05% Tween 20 (TBST)) and incubated with primary antibodies in the TBST in 4℃ overnight. Herein, the anti-ZO-1 antibody (Invitrogen), the anti-Occludin antibody (Invitrogen), the anti-Claudin 5 antibody (Invitrogen), the anti-vascular endothelial (VE)-Cadherin antibody (Abcam), and the anti-beta-Actin antibody (Senta Cruz) were utilized, respectively. After being washed three times with TBST, the membrane was probed with an HRP-conjugated secondary antibody and was developed with SuperSignal West Dura Extended Duration Substrate (Thermo Fisher) and imaged on an Image Quant TM LAS 4000.

### Microvascular endothelial cells monolayer resistance measurement

Cell resistance can reflect the function of the physiological barrier. In order to detect whether H7N9 virus infection could affect the function of BBB in vitro, Electric cell-substrate impedance spectroscopy (ECIS) technology was used to detect the resistance changes of hCMEC/D3 cells after infection. ECIS experimental procedure involved a pretreating 8W10E ECIS plate with 10 mM L-cysteine prior to coating with 3 µg/cm^2^ collagen I (Gibco). Epithelial cells were seeded on wells at a density of 4 × 10^4^/well, and the volume of culture medium for each well was 400ul. Cells were then infected H7N9 virus with different MOIs until a barrier had formed, typically ~ 60 h post-seeding. The endothelial barrier resistance was then monitored, at which point multi-frequency (ranging from 62.5 to 64,000 Hz) data was collected and modelled using ECIS software (Applied Biophysics).

### In vitro BBB permeability assay

The hCMEC/D3 cells were seeded at a density of 5 × 10^4^ per well on a Transwell insert (0.4 μm pore size, Corning) coated with 3 µg/cm^2^ collagen I in 100 µl complete ECM and 500 µl of the same medium were added to the basal chamber. Usually, confluent monolayer formation was assessed 48 h post-plating. To test whether the permeability of the barrier model was affected by H7N9 virus (MOI = 1), FITC-dextran (wt4000, Sigma) permeability was determined during the BBB exposure to H7N9 virus. The inserts were placed onto companion plates containing 500 µl of ECM. Before FITC-dextran was added to the media in the apical insert, the inserts were washed twice with prewarmed PBS. Then FITC-dextran was diluted to 100 µg/ml with ECM and added to the apical insert to incubate 30 min at 37℃, and 100 µl of ECM was collected from the basal companion wells. The fluorescence intensity of FITC-dextran was measured by a fluorescent plate reader (excitation 492 nm and emission 518 nm).

### Evans blue-BSA permeability assay

The Evans Blue (EB) was dissolved in PBS into 0.5% (w/v) solution, and then BSA was added to the EB solution to a concentration of 1% (w/v). After complete vortex mixing, let the solution stand for 30 min at room temperature and filter it with a 0.22 μm filter for later use. The hCMEC/D3 cells were seeded on a Transwell insert and infected by H7N9 virus as above. 2 h, 24 and 48 h after infection, EB-BSA was added to the apical insert to incubate at 37℃, and 50 µl of ECM was collected from the basal companion wells every 10 min until 50 min. After collecting the ECM, the basal was replenished every time. 0.5% EB solution was double-diluted to plot a standard curve simultaneously. The concentration of EB was measured by a microplate reader (OD620), and taking the time as abscissa and OD620 as the ordinate, the standard curve and the experimental group curve were plotted, respectively. The permeability coefficient (Pe) of EB-BSA was calculated by the following formula:

$$Pe=\frac{1}{1/{m}_{e}-1/{m}_{s}}\times \frac{1}{s}$$

where ms is the slope of the standard curve, and me is the slope of the curve in the experimental group. Moreover, s is the surface area of the inserts (1.12 cm^2^).

### Statistics analysis

Prism 8 (GraphPad) was used for data analysis and chart drawing. If the data of the two groups were compared, the student’s t-test was use to for statistical analyses. If more than two groups were compared, One-way ANOVA was used for statistical analyses. If *p* < 0.05, there is a statistical difference. Statistical significance is defined as, n.s., not significant, *p < 0.05, **p < 0.01, ***p < 0.001.

## Results

### H7N9 virus infects HCMEC/D3 cells without effect on cell viability

**Fig. 1:**
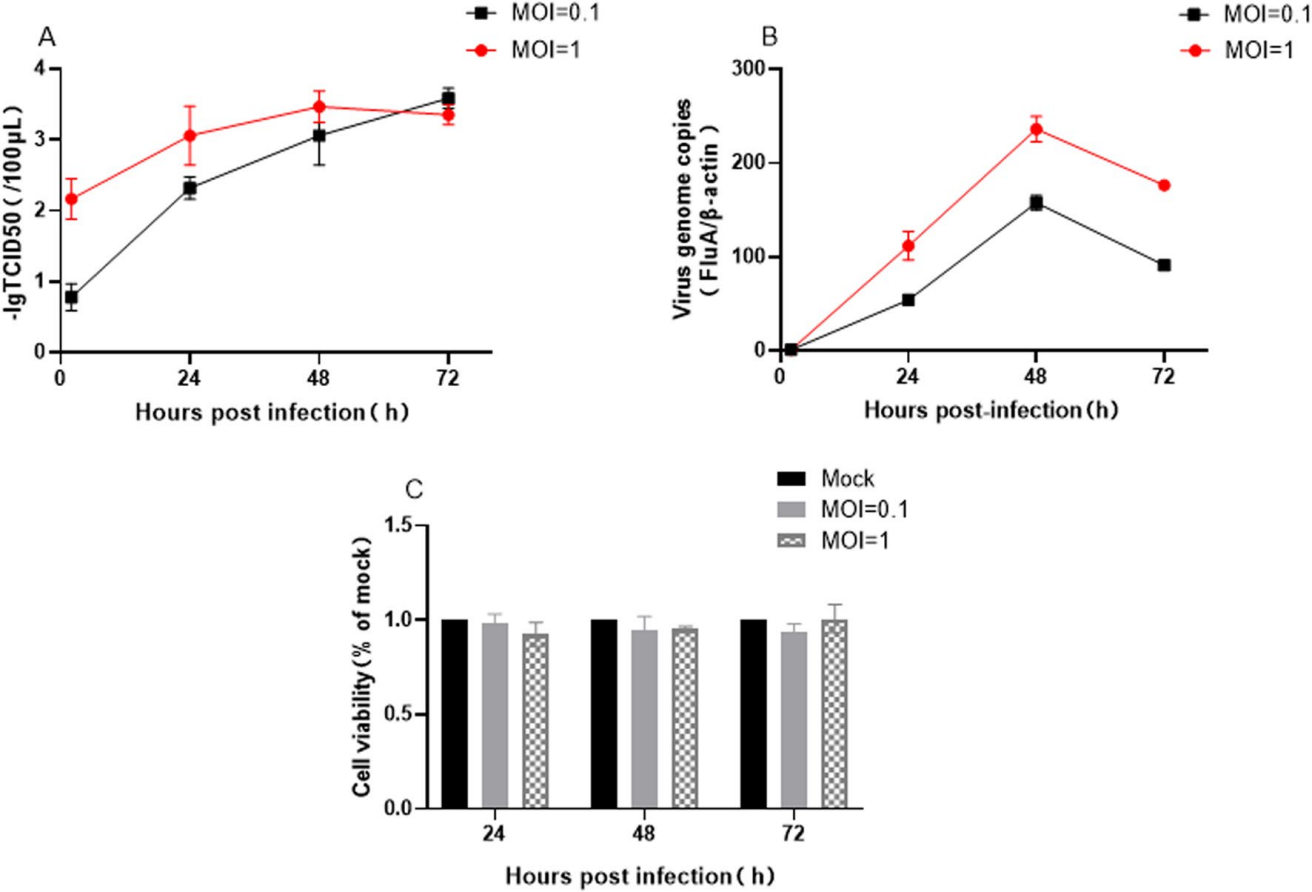
H7N9 influenza virus replicated in human brain microvascular endothelial cells hCMEC/D3 but did not affect cell viability during infection. **A** Virus replication in the cell supernatant after H7N9 influenza virus infected hCEMC/D3. **B** The viral nucleic acid amplification in cells after H7N9 influenza virus infected hCEMC/D3. **c** Changes in cell activity after H7N9 influenza virus infected hCEMC/D3

HCMEC/D3 cells are the immortalized human brain capillary endothelial cell line frequently employed in studies of BBB[30]. Here we used hCMEC/D3 cells as the cell model to investigate whether H7N9 virus could affect BBB in vitro. In both intracellular and supernatant, H7N9 viral genome and infectious progeny viruses were elevated over infection, indicating that H7N9 virus could infect hCMEC/D3 cells and produce progeny viruses (Fig. 1A, B). Furthermore, to investigate whether H7N9 virus caused a cytopathic viral infection, we detected the viability of hCEMC/D3 cells every 24 h during infection. Interestingly, CCK-8 results showed H7N9 virus infected cells but did not affect cell viability, even at the MOI of 1 at 72 h post-infection (Fig. 1C).

### H7N9 virus affects cell morphology on HCMEC/D3 cells

**Fig. 2:**
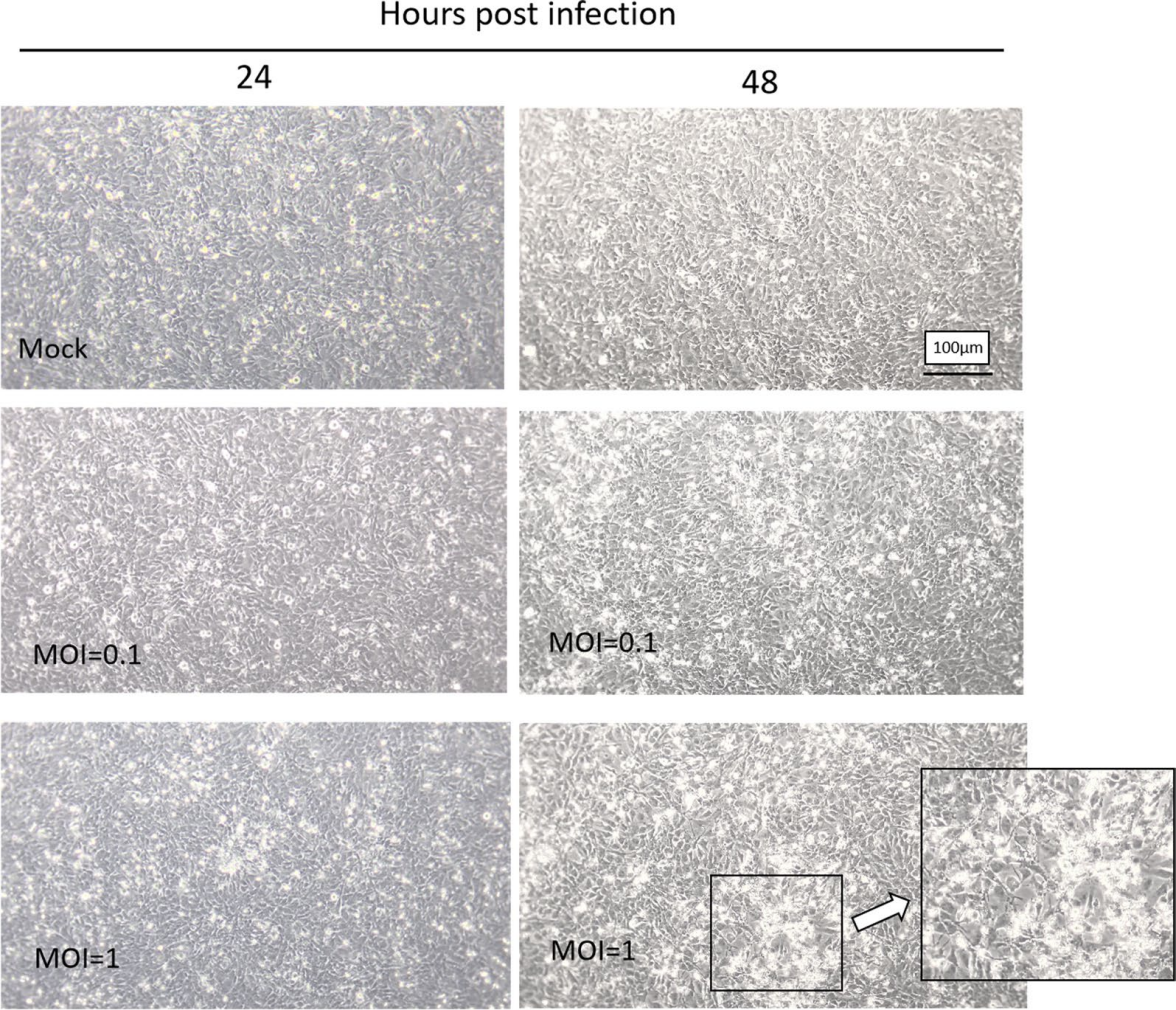
H7N9 influenza virus changed the cell morphology of hCMEC/D3 cells post-infection. The black box shows the morphological changes at MOI = 1 at 48hpi. At this point, the cells lost their original endothelial cell-like morphology and aggregated into clusters. Moreover, gaps appeared between nearby cells

Although H7N9 virus infection did not cause hCMEC/D3 cell death, we could not exclude cell morphological changes induced by infection. The results showed that at 24 h after infection, the hCMEC/D3 cells infected with H7N9 virus at the MOI of 1 initially appeared cell morphological changes. At 48 h post-infection, noticeable morphological changes were observed. Furthermore, intercellular gaps appeared near the cells that had lost their typical morphology. The infected cells appeared to lose the epithelial-cell-like morphology and shrink into clusters (Fig. 2). The cells did not present morphological changes until 48 h after infection at the MOI of 0.1. To hCMEC/D3 cells, although H7N9 virus infection did not affect cell viability, cell morphology changes might affect endothelial cells’ barrier function.

### H7N9 virus down-regulates the expression of adherens and tight junction proteins by affecting transcription

**Fig. 3:**
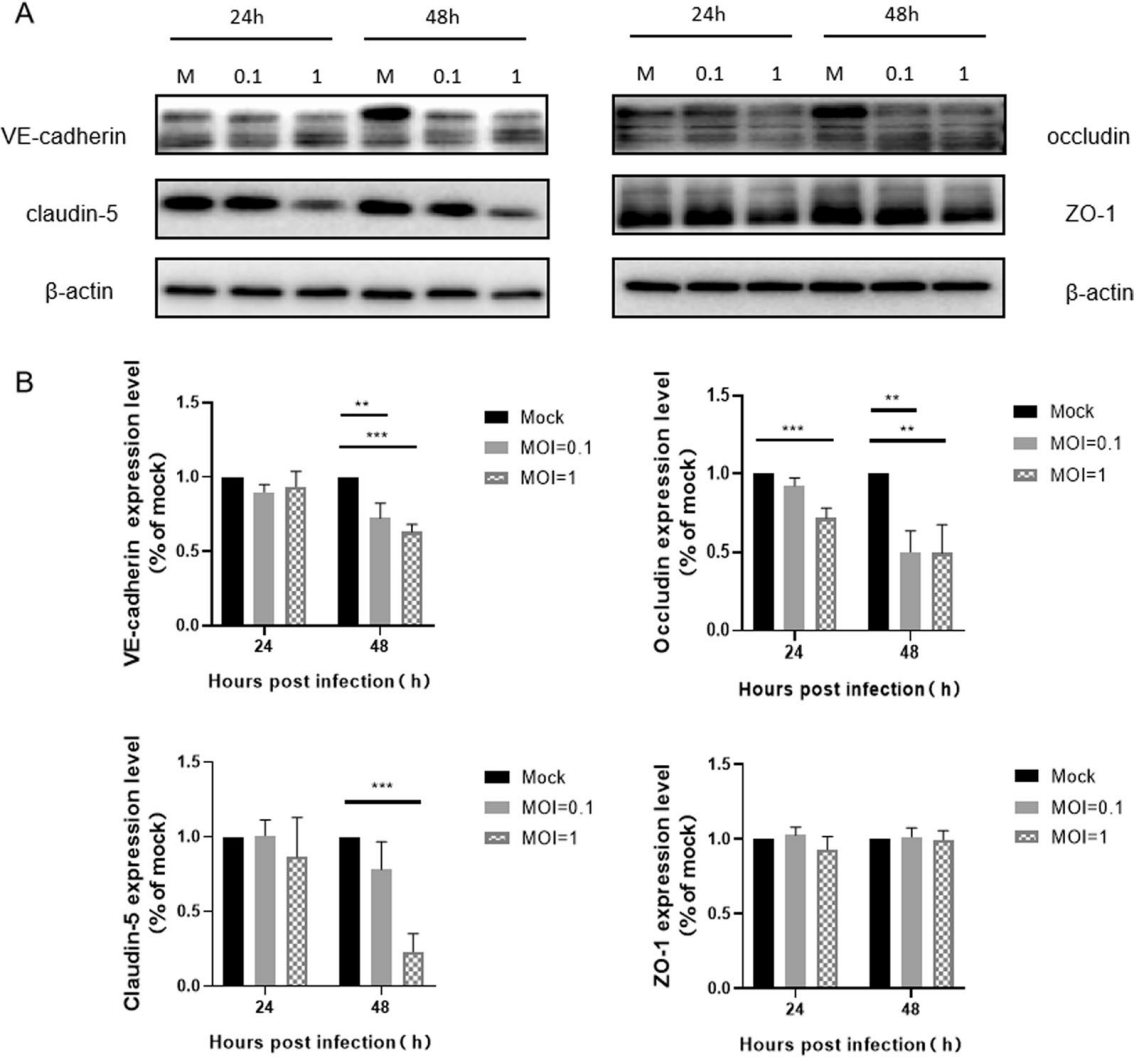
Influenza H7N9 virus infection decreased the expression of junctional proteins VE-cadherin, Occludin and Claudin-5 in hCMEC/D3 cells. **A** WB depicted that the amount of VE-cadherin, Occludin and Claudin-5 decreased in hCMEC/D3 cells post-infection. The amount of junctional proteins expression was normalized by β-actin. **B** The expression level of VE-cadherin, Occludin, Claudin-5 and ZO-1 compared to the mock group was quantified by ImageJ. Statistical differences were obtained through One-way ANOVA. **p < 0.01, ***p < 0.001

The two major junctional complexes holding brain endothelial cells together are adherens junctions (AJs) and tight junctions (TJs). The destruction of junctional complexes damages the blood-brain barrier, which is the key to barrier functions to prevent the paracellular spread of various pathogens, including viruses [31–33]. In order to study the effect of H7N9 infection on the expression of junctional complexes proteins in hCMEC/D3 cells, the expression of TJ proteins (claudin-5 and occludin) and AJ protein (VE-cadherin) in hCMEC/D3 cells was detected by western blotting (WB). Besides, the primary cytoplasmic actin-binding protein zonula occludens protein-1 (ZO-1) was detected because it connects the TJs to the cell cytoskeleton and plays an essential role in the interaction between TJs and AJs [34, 35]. The WB results showed the expression level of those proteins, including VE-cadherin, claudin-5, and occludin in infected hCMEC/D3 cells were significantly lower at 48 h post-infection with viral dose dependence compared to the mock group (Fig. 3A, Additional file 1). But the expression level of ZO-1 in infected cells was not significantly different compared to the mock group (Fig. 3B).

**Fig. 4:**
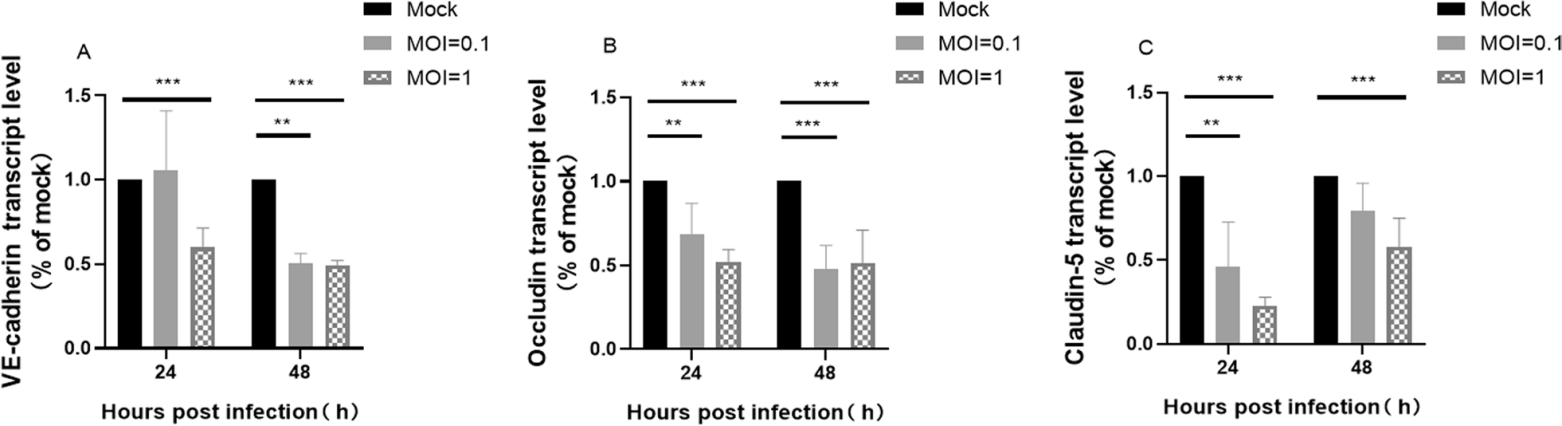
Influenza H7N9 virus infection decreased the transcript level of junctional proteins VE-cadherin, Occludin, and Claudin-5 in hCMEC/D3 cells. The number of transcripts was normalized by calculating 2^−ΔΔCt^. Statistical differences were obtained through One-way ANOVA. **p < 0.01, ***p < 0.001

Subsequently, qPCR detected the mRNA level of claudin-5, occludin, and VE-cadherin. The results showed that the mRNA level of all three proteins was down-regulated after H7N9 virus infection (Fig. 4). This suggested that H7N9 virus infection directly regulated the expression of tight junctional complexes proteins by regulating the transcriptional process.

### H7N9 virus reduces the monolayer hCMEC/D3 cells resistance

**Fig. 5:**
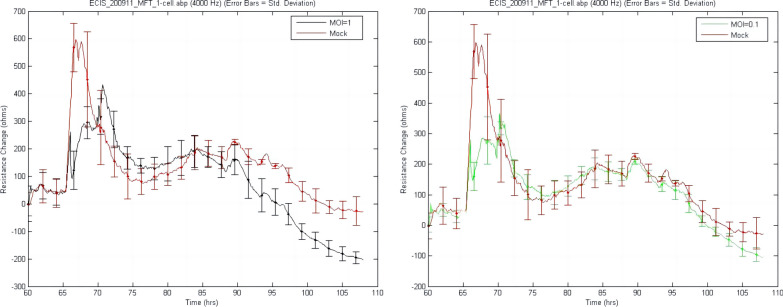
H7N9 influenza virus decreased the resistance of the infected hCMEC/D3 cell barrier. The cell seeding time is defined as 0 h. 60 h is approximately the time when the cells are fully grown, and the cell resistance reaches the plateau phase, so the X-axis start point is 60 h

Because of morphological changes and the destruction of cell junctions in infected hCMEC/D3 cells, we assumed the barrier function of endothelial cells was impaired. Cell resistance can reflect a cell barrier’s function[36]. Here ECIS system was used to detect the change in cell monolayer resistance during infection. After 36 h of infection, the resistance of cells with MOI of 1 was significantly lower than that in the mock group. However, no significant difference existed between infected cells with MOI of 0.1 and the mock group cells. Until 48 h post-infection, the resistances of both infected groups were significantly lower than the resistance of the mock group (Table 2). The resistance decreased continually in the group with MOI of 1, and the peak that appeared at 65 h was due to unstable fluctuations caused by the operation of changing medium when cells were infected with H7N9 virus (Fig. 5).

**Table 2:** The primers used in qPCR

Hours post infection (h)	The resistance of hCMEC/D3 cells (ohm)		
	Mock	MOI = 0.1	MOI = 1
0	819.80 ± 61.03	813.63 ± 16.30	815.33 ± 52.49
12	885.14 ± 71.32	889.38 ± 33.86	895.33 ± 42.09
24	1006.06 ± 67.11	980.73 ± 19.60	921.83 ± 52.01
36	772.30 ± 41.80	732.08 ± 20.85	627.57 ± 29.78***
48	680.92 ± 31.61	595.88 ± 12.90***	518.47 ± 14.91***

The table shows the resistance of monolayer hCMEC/D3 cells after H7N9 influenza virus infected at MOI of 0.1 and 1. Data are presented as mean ± standard deviation. The H7N9 infection groups were compared with the mock group. Statistical differences were obtained through One-way ANOVA. ***p < 0.001

### H7N9 virus increases the permeability of transwell model

**Fig. 6:**
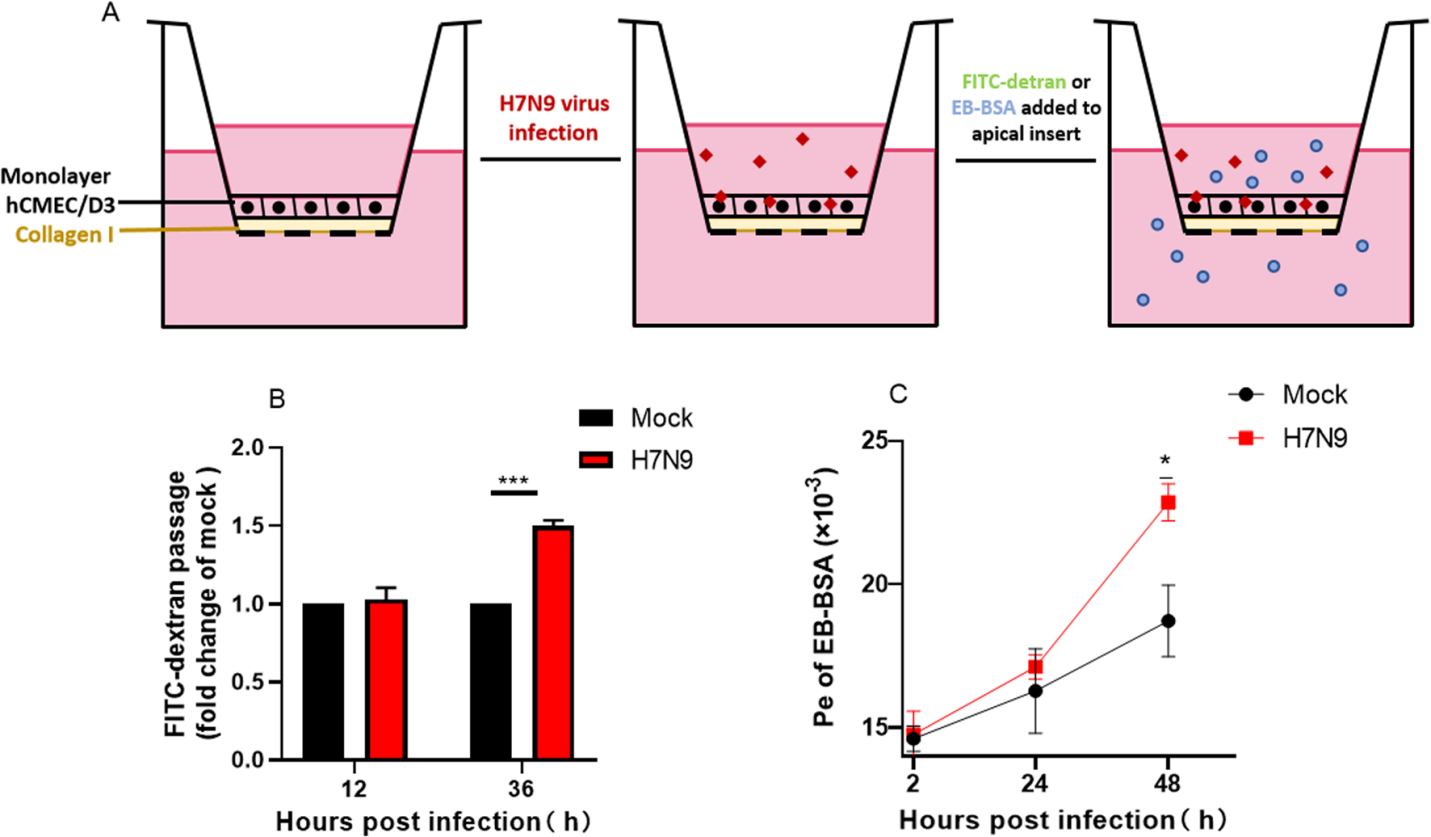
H7N9 influenza virus increased the permeability of the hCMEC/D3 cell barrier. **A** Schematic diagrams depict FITC-dextran or EB-BSA crossing the in vitro monolayer barrier cells model. **B** Changes of FITC-dextran permeability post-infection. **C** Changes of the Pe of EB-BSA post-infection. Statistical differences were obtained through t-tests. *p < 0.05, ***p < 0.001

In addition to the cell resistance, we also examined the barrier function of endothelial cells by constructing an in vitro BBB model to detect its permeability to the indicator FITC-dextran. BBB models in vitro have been used routinely to evaluate the mechanisms of therapeutic drugs and viruses across the BBB. We chose the mono-culture model for our experiment [30, 37]. To determine whether H7N9 Virus could affect the permeability of the BBB model in vitro, H7N9 virus was added into the apical chamber of the inserts containing hCMEC/D3 cells monolayer (Fig. 6A). Barrier function of the Transwell model was reacted with FITC-dextran permeability at 12 and 36 h post-infection. The results showed that the permeability of cells to FITC-dextran increased at 36 h after H7N9 virus infection compared with the mock group (Fig. 6B).

Besides FITC-dextran, EB-BSA was also used as an indicator to test the permeability of the in vitro model. Being different from the permeability of FITC-dextran, we represented the permeability of the in vitro model to EB-BSA by calculating Pe. The permeability of cells to EB-BSA increased at 48 h after H7N9 virus infection compared with the mock group (Fig. 6B), which was consistent with changes in the cell resistance and the FITC-dextran permeability, indicating that H7N9 infection could affect the barrier function of BBB in vitro.

## Discussion

Human infections with HPAI H7N9 virus cause primarily respiratory disease, but some populations, such as children, the elderly, and gravidas, are at risk of influenza-related central nervous system complications. However, the pathogenic mechanism of influenza viral encephalopathy has not been illustrated. In this study, we found that H7N9 influenza virus could disrupt the BBB function in vitro and increase its permeability by down-regulating the expression of junctional complex proteins. The present study provides new insight into the pathogenesis of H7N9 viral encephalopathy.

If H7N9 virus enters the CNS through the bloodstream, it must cross the BBB, a barrier between blood and brain tissue. Changes in the barrier characteristics are an essential reason for the pathology and progression of various neurological diseases [26–28, 38]. Here we used hCMEC/D3 as endothelial cells in the BBB to determine the infectivity and virulence of H7N9 virus on human brain microvessels endothelial cells, which is an important component of the BBB. The infection experiment has shown H7N9 virus could infect brain endothelial cells and produce infectious progeny viruses that did not cause cell death but affected the cell morphology during infection, which provides preliminary evidence that the H7N9 virus has the potential to infect the BBB.

Endothelial cell-cell connections are critical to barrier function, and two substructures of the junctional complexes that exist in the BBB participate in the cell connection, namely adherens junctions and tight junctions [27, 31]. The adherens junctions mediate cell-cell adhesion, and the tight junctions establish cell polarity and regulate the paracellular transport of ions and small molecules [34]. In this study, we found that H7N9 virus could infect human brain microvascular endothelial cells hCMEC/D3 and reduce the expression of cell junctional complexes proteins in cells. The transmembrane molecules TJ proteins, including claudins and occludins, are closely related to the intercellular junction. Claudin-5 is the most enriched TJ protein at the BBB [39], and mice that lack claudin-5 have a size-selective leak of the BBB [40]. Occludin is a tetraspanin strongly expressed at the interface of CNS endothelial cells [41], an in vitro culture experiment disrupting occludin homotypic interactions suggests that it is important for barrier resistance [26]. In infected hCMEC/D3 cells, the decreased expression of both claudin-5 and occludin indicated the effect of H7N9 virus infection on intercellular junctions of endothelial cells. Besides TJs, AJs are other major cell-to-cell connecting structures that sense and respond to tensile forces at the intercellular contact interface. The TJs interact with basal AJs, including the transmembrane protein VE-cadherin, which has five extracellular cadherin repeat domains mediating cell junctions [42]. VE-cadherin expression is restricted to the vascular system playing a crucial role in establishing AJs [43]. Consistent with TJ proteins results, VE-cadherin expression was down-regulated at 48 h after infection, resulting in disruption of cell junctions. As transmembrane adhesion complexes, the ZO proteins are cytoplasmic scaffolding proteins located in the cytoplasmic domain of TJs [31, 38]. Among ZO proteins family, ZO-1 plays a critical role in endothelial cell junctions by linking TJ proteins to the actin cytoskeleton and as a linkage between the AJs and TJs [26, 32, 44]. However, we did not find H7N9 influenza virus down-regulated the expression of ZO-1 in human brain microvascular endothelial cells, which indicated H7N9 virus disturbed the cell-cell contacts by directly down-regulating the transmembrane part of junctional complexes, rather than affecting the interaction with AJs and TJs components and the link between TJs and the cytoskeleton. We concluded H7N9 virus caused down-regulation of the expression of cell junction proteins, allowing the virus to disrupt the cell barrier. In addition, we detected the mRNA level of three down-regulated proteins, VE-cadherin, occludin, and claudin-5. The results showed that all proteins were lower than the mock control at the transcriptional level. This indicated that H7N9 virus infection directly reduced expression by affecting tight junction proteins’ transcription.

Based on the results above, we speculated that the infection of H7N9 virus would affect the normal physiological function of the BBB. In order to verify the phenomenon, we detected the cell resistance to see if the barrier was intact. ECIS has been applied to study BBB biophysical and biomedical functions including cell motility, wound healing and cell-cell adhesion [45, 46]. In this study, ECIS was used to detect cell resistance to reflect the integrity of the barrier function. With the infection of H7N9 virus, the resistance of monolayer hCMEC/D3 cells decreased gradually, which was consistent with the previous results. That is, the down-regulation of intercellular junction-related proteins affected the cell barrier function. In addition to the resistance detection of endothelial cells, we constructed an in vitro BBB model using Transwell inserts to verify the ECIS result. FITC-dextran is a bio-marker of the BBB permeability of high molecular mass molecules [47, 48], and the EB dye, also as a permeability marker, is often used to evaluate BBB integrity [49, 50]. Here they were used as indicators of whether the BBB function was impaired. Consistent with the electrical resistance test results, increased FITC-dextran and EB-BSA permeability in the infected model also demonstrated that H7N9 virus infection could cause in vitro BBB destruction. The destruction of the BBB increases the permeability of potentially neurotoxic molecules that may be harmful to the central nervous system and provides a way for H7N9 virus to enter the CNS.

## Conclusions

This study found that H7N9 virus infection caused cell morphological changes on hCMEC/D3 cells and down-regulated claudin-5, occludin, and VE-cadherin expression, which play critical roles in cell-to-cell contacts. Both the cell residence and the permeability illustrated H7N9 virus might infect the BBB and enter CNS by impairing the BBB. In addition, because H7N9 virus infection produced infectious progeny, the virus might also enter the CNS by intra-endothelial amplification. However, can H7N9 virus enter CNS in vivo and infect brain tissue to cause pathological changes? Does H7N9 virus cross the BBB through a paracellular or intracellular pathway like Zika virus[47]? More experiments are needed to answer these questions. In conclusion, our study provides clues as to whether H7N9 virus enters the CNS and a theoretical basis for the pathogenesis of H7N9 viral encephalopathy.

### Supplementary Information

**Additional file 1**. Original uncropped figures for blots.

## Data Availability

The datasets used and/or analyzed during the current study are available from the corresponding author on reasonable request.

## Abbreviations

VE: Vascular endothelial
NP: Nuclear protein
M: Matrix protein
IAV: Influenza A virus
CNS: Central nervous system
HPAI: Highly pathogenic avian influenza
BBB: Blood-brain barrier
ECM: Endothelial cell medium
FBS: Fetal bovine serum
P/C: Penicillin/streptomycin
DMEM: Dulbecco's modified eagle medium
MOI: Multiplicity of infection
BSL-3: Class III bio-safety lab
TCID50: 50% cell culture infectious dose
HA: Agglutination
TBST: TBS with 0.05% Tween 20
ECIS: Electric cell-substrate impedance spectroscopy
EB: Evans Blue
Pe: Permeability coefficient
AJs: Adherens junctions
TJs: Tight junctions
ZO-1: Zonula occludens protein-1
BSA: Bovine serum albumin
