# Supplementary figures and images for: Influenza H7N9 virus disrupts the monolayer human brain microvascular endothelial cells barrier in vitro
Source: Virol J. 2023 Sep 29;20:219. doi: 10.1186/s12985-023-02163-3 (PMC10541704; doi:10.1186/s12985-023-02163-3)

VE-cadherin

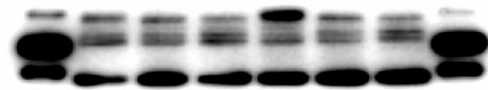

claudin-5

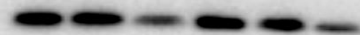

$\beta$ -actin

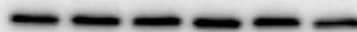

occludin

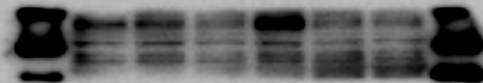

ZO-1

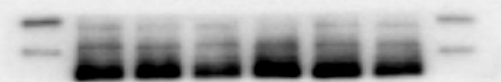

$\beta$ -actin

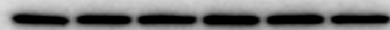

Supplement: Supplementary file 1 — Additional file 1. Original uncropped figures for blots. [file 12985_2023_2163_MOESM1_ESM.pdf]
